# Effect of verjuice (*Vitis vinifera* L.) on physicochemical and textural properties of beef *M. biceps femoris*

**DOI:** 10.1002/fsn3.4192

**Published:** 2024-05-07

**Authors:** Fereshteh Sabzi, Mohammad Javad Varidi, Mehdi Varidi, Maryam Asnaashari

**Affiliations:** ^1^ Department of Food Science and Technology College of Agriculture, Ferdowsi University of Mashhad Mashhad Iran; ^2^ Innovative Medical Research Center, Faculty of Medicine, Mashhad Medical Science Islamic Azad University Mashhad Iran; ^3^ Department of Animal Processing Animal Science Research Institute of Iran (ASRI), Agricultural Research, Education and Extension Organization (AREEO) Karaj Iran

**Keywords:** beef *biceps femoris*, structure, tenderization, texture, verjuice

## Abstract

The objective of this study was to investigate the effect of verjuice on beef *M. biceps femoris* (BF). BF blocks were marinated with 30%, 70%, and 100% verjuice solutions containing 2% NaCl, for different marination times (12, 24, 48, and 72 h). Verjuice marination reduced the pH values of BF samples from 6.77 in control sample to 3.66 in 100% of verjuice for 72 h. The decreased values of water holding capacity (from 54.06% to 47.46%) with increasing verjuice concentration (from 30% to 100% for 72 h) confirmed the drop of proteins isoelectric point of the muscle due to salt presence preventing fibers swelling. Less cookout was observed with increasing acid concentration. Marination time had no significant effect on L* and a* coordinates of uncooked samples while acidification made the samples lighter and less red. Enzymatic proteolysis of myosin and troponin‐T concomitant with increase in myofibrillar fragmentation index contributed to the decrease of shear force in a way dependent on verjuice concentration and marination time. Sensory panelists gave the highest score to cooked samples marinated with 70% verjuice solution.

## Graphical abstract

The tenderizing effect of verjuice with different concentrations on muscle quality of beef *M. biceps femoris* (BF). The obtained results showed that verjuice solutions can be utilized to ensure a more desirable texture of BF muscle. The abundance, low price, and most importantly the occurrence of polyphenolic compounds in verjuice make it a better and affordable replacer for chemical tenderizers or even herbal proteases.

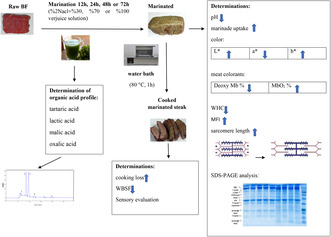

## 1 INTRODUCTION

Meat tenderness is considered as one of the most important qualitative parameters in consumer acceptance (McCormick, 1994). There are numerous factors affecting meat tenderness, the most important of which are the myofibrillar protein components of the muscle (Lawrie, 1998), as well as the content and solubility of the connective tissue, especially collagen (Torrescano et al., 2003).

Beef *M. biceps femoris* (BF), a collagen‐rich muscle, is generally hard; that is why, its tenderness is not accepted by consumers. Nowadays, meat producers and researchers are trying to improve the quality and overall acceptability of BF using numerous tenderization techniques including enzymatic, mechanical, and chemical methods. Application of suitable methods for meat tenderization can be helpful in the modification of textural properties, improvement of its edible quality and juiciness, and the reduction in cooking loss and tenderization time in refrigerator (Alvarado & McKee, 2007).

Since the tenderness and water‐holding capacity of beef enhance under acidic conditions lower than the postmortem pH (pH 5.2–5.5) (Berge et al., 2001), the immediate postmortem pH reduction of meat through acid marinating has been suggested as a practical method to improve its technological and functional properties, such as tenderness, water holding capacity, flavor, and odor (Yusop et al., 2010). In addition, the increment of product shelf‐life would be achieved through inhibiting bacterial growth, especially pathogens (Pathania et al., 2010). In different studies, various acids and acidic materials, including lactic acid, citric acid, acetic acid, ascorbic acid (Aktaş et al., 2003; Berge et al., 2001; Burke & Monahan, 2003; Hinikle, 2010; Ke et al., 2009; Sultana et al., 2008), soy sauce (Kim et al., 2013), orange juice (Latif, 2011), pomegranate fruit (Narsaiah et al., 2011), kefir, lemon juice, wine, pineapple (Xiong et al., 2002; Żochowska‐Kujawska et al., 2012), mustard‐honey, apple vinegar, white wine vinegar and kefir (Tänavots et al., 2018), leek (*Allium ampeloprasum*) extract (Mehrabani et al., 2022), papain, bromelain and Zingiber officinale (Habtu et al., 2020), cashew fruits (*Anacardium occidentale*) (Ahmad et al., 2020), pineapple (*Ananas comosus*) and jackfruit (*Artocarpus heterophyllus*) by‐products (Ramli et al., 2021), have been already used to improve meat tenderization.

Three mechanisms have been suggested for meat tenderization by acidic solutions: (i) Meat acidification is accompanied by repulsion among filaments as a result of disrupting the electrical equilibrium of charges following an increase in positive charges. The weakness of meat structure and its swelling owing to water diffusion into it are among the consequences of this phenomenon; (ii) Acidic condition within the prerigor mortis step provides an appropriate environment for the activity of cathepsins leading to a more considerable proteolysis of myofibrillar proteins; and (iii) low pH causes a higher content of collagen to be converted into gelatin during cooking (Kim et al., 2013).

Verjuice is the juice of the unfermented green unripe grapes obtained via direct squeezing. Verjuice has a low pH (3.0–3.5) and unique sour flavor which is used in Mediterranean countries, southeast of Türkiye, and different regions of Iran as a replacement for vinegar and lemon juice for preparing salad dressings and appetizers. It is also employed as an ingredient in various drinks, sausages, and mustard. The major acids of verjuice are tartaric and malic acid. Other acids, including caffeic, caftaric, fertaric, gallic, 2‐*S*‐glutathionyl caffeoyl tartaric (GRP), coumaric, coutaric, and protocatachuic acid, have also been measured in verjuice (Nikfardjam, 2008).

With regard to the acidic property of verjuice, it is expected to have a dramatic effect on meat tenderness. The objectives of this study were to investigate the tenderizing effect of verjuice with different concentrations on BF and to monitor the effect of marinating time in verjuice solution on physicochemical and textural properties of the respective muscle.

## 2 MATERIALS AND METHODS

### 2.1 Preparation of the muscle samples and marinades

Four *biceps femoris* (BF) beef muscles from four different cows (approximately 2–3 years old with the same diet) were purchased from an industrial slaughterhouse in Mashhad (Iran) at 24 h postmortem. The study was performed with four replicates (*n* = 4) and each muscle from a cow was considered as an experimental replicate. After removing visible connective tissue and external fat, the center portion of each muscle was cut into rectangular cubes with the dimensions of 3 × 15 × 15 cm (approximately 200 g). The cubes were randomly devoted to four treatments (16 cubes per each treatment).

Verjuice was supplied by Golchekan Zamani company (Mashhad, Iran). Marinades of 30% (30 verjuice: 70 distilled water), 70% (70 verjuice: 30 distilled water), and 100% (100 verjuice: 0 distilled water) were prepared and their pH was recorded. A verjuice‐free treatment (distilled water) was considered as the control. NaCl concentration was kept constant in all marinades (2% w/w).

### 2.2 Verjuice injection into the samples and their marination

The weight of all prepared meat pieces was recorded (A&D Weighing, GF‐600 Precision Scale, Japan) and verjuice solutions of 30%, 70%, and 100% were injected into each sample as much as 10% of its initial weight in 1‐cm intervals, using a special injector (BQ Meat Injector, JBQ010, China). Then, the samples were marinated in the respective solutions for 12, 24, 48, and 72 h at 4°C to increase the efficiency and uniform distribution of verjuice solutions throughout all surface and deep parts of the meat. The ratio of verjuice solution to meat was regarded as 4–1. In different time intervals, the samples were removed from the solutions and weighed again after blotting the excess solution. At the same time, a verjuice‐free sample was taken into account as the control sample for each time treatment. Therefore, the study was performed with four replicates. The factors studied were verjuice concentration (0%, 30%, 70%, and 100%) and marination time (12, 24, 48, and 72 h).

### 2.3 Analyses

#### 2.3.1 pH

The pH of the samples was measured using a probe pH meter (Testo 230, Germany). pH measurements were carried out by placing the probe on 5 certain points of the control and marinated samples in the depth of 1.5 cm and the average was reported as the final pH.

#### 2.3.2 Marinade uptake

The marinade uptake of each sample was determined by the following equation (Kim et al., 2013):

(1) $$\text{Marinade uptake}\;\left(\%\right)=\frac{W_{2}-W_{1}}{W_{1}}\times 100$$

where *W*_1_ and *W*_2_ are the sample weight before and after marination (g), respectively.

#### 2.3.3 Color

The color parameters (L*, a*, and b*) corresponding to the surfaces of treated samples were measured using a digital Chroma meter (CR‐410, Konica Minolta sensing, Japan) with a 50‐mm aperture, a C illuminant, and a 2° standard observer. The apparatus was calibrated using a white tile with the color parameters of L* = 98.14, a* = − 0.23, and b* = 1.89.

#### 2.3.4 Meat colorant measurements

Total myoglobin (Mb) content and the related pigments including oxymyoglobin (MbO_2_), deoxymyoglobin (deoxyMb), and metmyoglobin (MetMb) were measured spectrophotometrically following their extraction (Krzywicki, 1982) with some modification. Briefly, 2‐g beef turned into homogenized with 20 mL of .04 mM phosphate buffer, pH 6.8, the use of an Ultra‐Turrax T25 homogenizer (IKA LABORTECHNIK) at 10,000 g and room temperature for 20 s. After resting for 1 h in an ice bath, the homogenate was centrifuged (10,000 g for 30 min at 10–15°C). The supernatant was filtered via Whatman No. 1 paper and the volume was brought up to 25 mL with the same phosphate buffer. Turbidity was avoided by maintaining a low temperature and by extra filtration with the use of a 0.25‐μm Millipore filter prior to spectrophotometry measurement, as necessary. The absorbance of diluted filtrate obtained from the supernatant of homogenized samples in .04 mM phosphate buffer pH 6.8 was measured at 525, 545, 565, and 572 nm. Total Mb content and the percent of MbO_2_, deoxyMb, and MetMb were calculated using the following equations:

(2) $$\begin{array}{c} \text{Total myoglobin}\;\left(\mathrm{mM}/L\right)=–0.166\;A_{572}+0.086\;A_{565}\\ \;+0.088\;A_{545}+0.099\;A_{525} \end{array}$$

(3) $$\%\text{deoxyMb}=\left(0.369\;R_{1}+1.140\;R_{2}–0.941\;R_{3}+0.015\right)\times 100$$

(4) $$\%{\mathrm{MbO}}_{2}=\left(0.882\;R_{1}–1.267\;R_{2}+0.809\;R_{3}–0.361\right)\times 100$$

(5) $$\%\text{MetMb}=\left(–2.541\;R_{1}+0.777\;R_{2}+0.800\;R_{3}+1.098\right)\times 100$$

where *R*_1_, *R*_2_, and *R*_3_ are respectively, the absorbance ratio *A*_572_/*A*_525_, *A*_565_/*A*_525_, and *A*_545_/*A*_525_.

#### 2.3.5 Water‐holding capacity (WHC)

WHC of samples was determined according to Sultana et al. (2008). 0.3 g of meat was placed on Whatman filter papers (No. 1) between two Plexiglass plates. A 2‐kg body was then put on the plate for 5 min. The meat sample separated from the filter paper was weighed and WHC was calculated as follows:

(6) $$\text{Water Holding Capacity}\%=1-\left[\frac{W_{1}-W_{2}}{W_{1}-M_{c}}\right]\times 100$$

where *M*_C_ stands for the sample water content. *W*_1_ is the initial weight of the sample (before pressing) and *W*_2_ represents the final weight of the sample (after pressing).

#### 2.3.6 Cooking loss

Fifty‐gramloaves placed in polyethylene bags were cooked in a water bath until their internal temperature reached 71°C. The cooled samples were blotted and weighed (Kim et al., 2013). Cooking loss was determined as follows:

(7) $$\text{Cooking loss}\%=\frac{W_{1}-W_{2}}{W_{1}}\times 100$$

where *W*_1_ and *W*_2_ are the sample weights (g) before and after cooking, respectively.

#### 2.3.7 Warner–Bratzler shear force (WBSF)

The required force for cutting samples (WBSF) was measured using the TA.Xtplus texture analyzer (Stable Micro Systems, England) and according to the method suggested by Byrne et al. (2000). The cooked samples cut in parallel to the muscle fiber with a diameter of 1.25 cm were placed in the apparatus so that the Warner–Bratzler blade cut them vertical to muscle fibers with the speed of 200 mm/min.

#### 2.3.8 Myofibrillar fragmentation index (MFI)

MFI was determined according to Culler et al. (1978) with slight modification. The ground meat homogenized in cold MFI buffer pH 7 (100 mM KCl, 20 mM K phosphate, 1 mM EDTA, 1 mM MgCl_2_ 1 mM NaN_3_) was centrifuged at 1000 × *g* for 15 min. Following the resedimentation of the pellet with the same volume of buffer, the new pellet was mixed with buffer and passed through mesh strainers with 1 mm^2^ holes to remove connective tissue and debris. The protein concentration of fibrils was determined by the Biuret method. An aliquot of the myofibril suspension was diluted with isolating medium (MFI buffer) to a protein concentration of 0.5 mg/mL and then the protein concentration of the suspension was again measured by the Biuret method (Gournall et al., 1949).

#### 2.3.9 Sarcomere length

The meat pieces stored in 2.5% glutaraldehyde A (2.5% glutaric di‐aldehyde, 0.1 M KCl, 0.039 M H_3_BO_3_, 5 mM Tritriplex III) and 2.5% glutaraldehyde B (2.5% glutaric di‐aldehyde, 0.25 M KCl, 0.29 M H_3_BO_3_, 5 mM Tritriplex III) were homogenized in the latter and a drop was placed between a slide and a cover slip (Botha et al., 2007). Microscopic images were captured with the magnification of ×100 using a light microscope (BH_2_, Olympus, Japan) connected to a digital camera (DXM‐1200, Nikon, USA). The images were analyzed using Adobe Photoshop, v. 6.0 (Adobe, San Jose, CA, USA).

#### 2.3.10 Separation of the myofibrillar proteins

Separation of myofibrillar proteins was accomplished based on the method of Claeys et al. (1995). The mixture of 25 mL of buffer solution (0.25 M sucrose, 0.05 M Tris–HCl, and 1 mM EDTA, pH 7.6, 3°C) and 2.5 g of the ground meat was homogenized at 16,000 rpm for 30 s and then centrifuged at 1000 × *g* for 10 min. The pellet of previous step was mixed with 25 mL of buffer solution (0.05 M Tris–HCl, 1 Mm EDTA, pH 7.6, 3°C) and resedimented. In the third step of extraction, 25 mL of 0.15 M KCl was utilized. The final pellet which is myofibrillar proteins was mixed with 10 mL of the cold MFI buffer (4°C) and stored in a fridge until SDS‐PAGE electrophoresis (Culler et al., 1978).

#### 2.3.11 SDS–PAGE analysis

SDS–PAGE analysis was done in accordance with the procedure explained by Laemmli (1970). Proteins were separated on 4% stacking and 12% separating gels using a Mini‐protean Tetra Cell (Bio‐Rad, USA). For this purpose, 50 μL of the protein isolate solution with a given concentration was mixed with 150 μL of the sample buffer comprising 2‐mercaptoethanol and SDS and then 40 μL of the prepared sample was injected into each well. Electrophoresis process was run at a constant voltage of 120 for 1.5–2 h. The applied ladder was also purchased from Sigma‐Aldrich (M4038) with a molecular weight range of 6.5–205 kDa. The resulting gels were stained using 0.125% Coomassie Brilliant Blue G_250_, 40% ethanol, and 10% acetic acid for 24 h. Destaining of the gels was done with 1% acetic acid. Analysis of the formed bands was performed by TotalLab TL_120_ software version 2009 (Nonlinear Dynamics Ltd, UK) through which some specifications including the band area, band percentage, and molecular weight were determined.

#### 2.3.12 Sensory evaluation

The marinated samples in different concentrations of verjuice solution (0%, 30%, 70%, and 100%) for 48 h were used for the sensory evaluation. For this purpose, 20 small pieces of each sample with the dimensions of 10 × 10 × 2.5 cm were put on an aluminum foil and cooked in a hot‐air oven (Memmert, Universal model, Germany) at 120°C. When the cold point temperature reached 72°C, the cooked pieces were cut in parallel to the muscle fibers, and a number of parameters including color, odor, flavor, tenderness, juiciness, and overall acceptability were evaluated at the sensory evaluation laboratory in individualized booths by 20 trained panelists (10 men and 10 women from Ferdowsi University of Mashhad, Mashhad, Iran). Each panelist evaluated all four treatments using a 5‐point hedonic scale. Bottled water was provided to clean the mouth between samples. In order to eliminate the time effect, sensory evaluation was carried out in one session.

### 2.4 Reverse‐phase high‐performance liquid chromatography

The quantity of some organic acids in verjuice (oxalic, tartaric, malic, and lactic acids) was measured using a RP‐HPLC (HPLC system VARIAN, Pro Star, USA) equipped with an ion exchange column (Aminex® HPX‐87H) a model 210 pump and diode array detector. The mobile phase was 0.01 N sulfuric acid (pH 2.27). For analysis, sample was prepared with a concentration of 5 g in 100 mL of mobile phase, filtered through a 0.45‐μm membrane, and then injected through model 410 autosampler.

The standard organic acids were precisely weighed and dissolved in deionized water. A set of serial dilutions of each organic acid was prepared. The concentration ranges of standard solutions were 0.001–500 mg/L for oxalic acid, 0.001–500 mg/L for malic acid and lactic acid, and 0.005–1000 mg/L for tartaric acid. Then, a 10‐μL aliquot of each serial dilution was injected into the HPLC system.

The validation parameters obtained from methods with standard solutions and spiked samples were the calibration curve linearity, recovery, and limit of detection (LOD). The linearity range was determined by injecting different concentrations obtained by the dilution of standard organic acids (tartaric, oxalic, malic, and lactic acid) in ultrapure water. Analytical curves for each organic acid were obtained considering the correlation between the peak area and the respective concentration of the standard solution. The recovery was calculated by comparing the result obtained analytically for each organic acid with the initial concentration in the spiked sample. Three standards were prepared at concentrations close to the estimated LOD and analyzed in triplicate. An analytical curve was constructed by plotting the values obtained from the analysis of the standards versus the actual values. The LOD of each organic acid was established as three times the RSD, respectively, added to the intercept of the curve.

### 2.5 Statistical analysis

This study was carried out using a full factorial completely randomized design. Verjuice concentration at levels of 0%, 30%, 70%, and 100% as well as marination time at levels of 12, 24, 48, and 72 h were analyzed in terms of their main and interaction effects on physicochemical properties of samples. The study was performed with four replicates. To investigate the effect of verjuice concentration on sensory parameters, a completely randomized design was applied. The obtained results were analyzed using the SPSS 16.0 software and the mean comparison was performed by Duncan's test with a confidence level of 95% (*p* < .05).

## 3 RESULTS AND DISCUSSION

### 3.1 Chemical and physicochemical properties of BF marinated with verjuice

All chemical, physicochemical, and textural properties measured for raw BF are summarized in Table 1. These findings are in line with results reported by Istrati et al. (2015). The main and interaction effects of verjuice concentration and marination time on these properties are presented in Tables 2 and 3, respectively.

**TABLE 1:** Physicochemical and textural properties of raw *biceps femoris* beef samples.

Traits^1^	Mean ± SD	Traits	Mean ± SD
Moisture (%)	77.32 ± 0.54	L*	32.121 ± 0.078
Protein (%)	18.32 ± 0.10	a*	22.751 ± 0.056
Fat (%)	5.36 ± 0.05	b*	6.950 ± 0.081
Ash (%)	0.098 ± 0.001	MFI	40 ± 2.828
pH value	6.840 ± 0.021	Sarcomere length (μm)	2.117 ± 0.087
WHC (%)	35.25 ± 0.280	WBSF (Newton)	80 ± 0.283
Cooking loss (%)	32.122 ± 2.15		

Abbreviations: MFI, myofibrillar fragmentation index; WBSF, Warner–Bratzler shear force; WHC, water‐holding capacity.

^1^ Values are mean ± standard deviation in two replicates.

**TABLE 2:** The main effects of verjuice concentration (a) and marination time (b) on physicochemical and textural properties of *biceps femoris* beef muscles.

(a)				
Properties of treated BF^1^	Verjuice concentration %			
	0	30	70	100
pH	5.97 ± 0.05^a^	5.29 ± 0.07^b^	5.04 ± 0.08^c^	4.27 ± 0.04^d^
Marinade uptake %	0.00 ± 0.00^b^	9.57 ± 0.25^a^	9.18 ± 0.38^a^	10.05 ± 0.42^a^
L*	42.45 ± 1.11^b^	50.55 ± 2.48^a^	52.86 ± 2.73^a^	51.91 ± 3.28^a^
a*	19.91 ± 1.04^a^	8.37 ± 0.55^b^	7.10 ± 0.18^b^	7.26 ± 0.20^b^
b*	8.12 ± 0.62^ab^	7.50 ± 0.74^b^	10.89 ± 1.10^a^	10.49 ± 0.64^ab^
WHC %	42.35 ± 0.85^d^	53.30 ± 0.56^a^	48.29 ± 0.84^b^	46.11 ± 1.00^c^
Cooking loss %	34.68 ± 2.66^b^	43.78 ± 1.57^a^	42.42 ± 2.83^a^	40.54 ± 2.93^ab^
MFI	51.75 ± 7.74^d^	59.50 ± 7.31^c^	64.75 ± 4.77^b^	67.00 ± 4.78^a^
Sarcomere length (μm)	2.66 ± 0.20^a^	2.51 ± 0.39^b^	2.63 ± 0.17^ab^	2.59 ± 0.28^ab^
WBSF (N)	72.65 ± 4.90^a^	69.17 ± 3.07^b^	53.20 ± 7.22^c^	44.67 ± 11.41^d^

(b)				
Properties of treated BF	Marination time (h)			
	12	24	48	72
pH	5.89 ± 0.04^a^	5.15 ± 0.04^b^	5.05 ± 0.05^c^	4.48 ± 0.06^d^
Marinade uptake %	4.84 ± 0.35^b^	7.25 ± 0.25^ab^	7.10 ± 0.54^ab^	9.61 ± 0.62^a^
L*	48.67 ± 7.29^a^	49.94 ± 8.64^a^	50.35 ± 8.82^a^	48.80 ± 9.57^a^
a*	10.80 ± 0.66^a^	11.19 ± 0.65^a^	10.33 ± 0.60^a^	10.32 ± 0.59^a^
b*	9.18 ± 0.16^a^	8.54 ± 0.19^a^	9.48 ± 0.18^a^	9.82 ± 0.17^a^
WHC %	47.20 ± 3.81^b^	47.39 ± 4.18^b^	47.36 ± 4.28^b^	48.11 ± 4.85^a^
Cooking loss %	37.81 ± 4.23^a^	40.87 ± 2.66 ^a^	42.92 ± 3.89^a^	39.81 ± 3.20^a^
MFI	53.00 ± 8.81^c^	59.75 ± 5.90^b^	61.50 ± 5.73^b^	68.75 ± 5.23^a^
Sarcomere length (μm)	2.57 ± 0.21^b^	2.48 ± 0.24^b^	2.54 ± 0.34^b^	2.80 ± 0.19^a^
WBSF (N)	67.79 ± 9.35^a^	62.13 ± 11.19^b^	56.91 ± 13.53^c^	52.84 ± 16.61^d^

Abbreviations: MFI, myofibrillar fragmentation index; WBSF, Warner–Bratzler shear force; WHC, water‐holding capacity.

^1^ Values are mean ± standard deviation.

**TABLE 3:** The interaction effects of marination time and verjuice concentration on physicochemical and textural properties of *biceps femoris* beef muscles.

Sample^1^										
	pH value	Marinade uptake (%)	L*	a*	b*	WHC (%)	Cooking loss (%)	MFI	Sarcomere length (μm)	WBSF (N)
No verjuice, 12 h	6.77 ± 0.04^a^	–	40.00 ± 0.20^a^	20.66 ± 0.10^a^	8.33 ± 0.07^ab^	43.54 ± 0.32^g^	32.44 ± 4.01^a^	41.00 ± 1.41^j^	2.45 ± 0.02^cde^	78.58 ± 4.36^a^
30% verjuice, 12 h	6.33 ± 0.05^b^	6.94 ± 0.64^ab^	48.20 ± 0.27^a^	8.66 ± 0.01^a^	8.05 ± 0.09^ab^	52.88 ± 0.73^a^	39.52 ± 3.80^a^	50.00 ± 0.00^i^	2.42 ± 0.14^cde^	73.46 ± 3.10^ab^
70% verjuice, 12 h	6.14 ± 0.05^b^	6.40 ± 0.23^ab^	55.48 ± 0.32^a^	7.05 ± 0.16^a^	10.67 ± 0.09^ab^	47.38 ± 0.66^cd^	40.02 ± 3.10^a^	59.00 ± 1.41^gh^	2.51 ± 0.09^bcde^	58.91 ± 3.65^cd^
100% verjuice, 12 h	4.32 ± 0.08^gh^	6.03 ± 0.07^ab^	48.07 ± 0.37^a^	6.82 ± 0.01^a^	9.63 ± 0.10^ab^	44.99 ± 0.49^f^	39.28 ± 2.91^a^	62.00 ± 0.00^defg^	2.45 ± 0.04^cde^	60.20 ± 9.09^cd^
No verjuice, 24 h	5.80 ± 0.08^c^	–	46.90 ± 0.60^a^	20.86 ± 0.29^a^	8.08 ± 0.16^ab^	42.48 ± 0.04^gh^	32.56 ± 0.53^a^	51.00 ± 1.41^i^	2.53 ± 0.10^bcde^	74.50 ± 9.55^ab^
30% verjuice, 24 h	5.21 ± 0.01^d^	9.07 ± 0.14^ab^	50.77 ± 0.39^a^	8.91 ± 0.07^a^	7.35 ± 0.17^ab^	53.16 ± 0.21^a^	43.77 ± 3.02^a^	60.00 ± 2.83^fg^	2.13 ± 0.01^e^	68.31 ± 19.24^abc^
70% verjuice, 24 h	4.86 ± 0.01^ef^	8.80 ± 0.38^ab^	50.46 ± 0.07^a^	7.03 ± 0.04^a^	9.64 ± 0.03^ab^	48.23 ± 0.06^c^	44.72 ± 4.65^a^	63.00 ± 1.41^defg^	2.67 ± 0.22^abcd^	58.25 ± 5.05^cde^
100% verjuice, 24 h	4.73 ± 0.01^f^	11.15 ± 0.17^a^	51.67 ± 0.07^a^	7.94 ± 0.08^a^	9.10 ± 0.25^ab^	45.68 ± 0.15^ef^	42.43 ± 3.47^a^	65.00 ± 1.41^cdef^	2.61 ± 0.09^abcd^	47.46 ± 21.84^ef^
No Verjuice, 48 h	5.67 ± 0.02^c^	–	42.38 ± 0.12^a^	18.91 ± 0.12^a^	7.66 ± 0.10^ab^	41.95 ± 0.27^h^	40.56 ± 1.97^a^	54.00 ± 0.00^hi^	2.86 ± 0.17^abc^	68.99 ± 16.50^abc^
30% verjuice, 48 h	5.15 ± 0.03^d^	11.38 ± 0.71^a^	52.13 ± 0.23^a^	8.48 ± 012^a^	8.83 ± 0.05^ab^	53.09 ± 0.18^a^	45.70 ± 2.78^a^	59.00 ± 1.41^gh^	2.15 ± 0.14^e^	69.02 ± 12.49^abc^
70% verjuice, 48 h	5.02 ± 0.01^de^	9.22 ± 0.73^ab^	54.53 ± 0.60^a^	6.92 ± 0.03^a^	13.04 ± 0.05^ab^	48.08 ± 0.64^c^	44.18 ± 3.04^a^	66.00 ± 0.00^bcde^	2.83 ± 0.04^abc^	56.62 ± 16.37^de^
100% verjuice, 48 h	4.34 ± 0.04^g^	7.22 ± 0.36^ab^	52.36 ± 0.06^a^	7.00 ± 0.11^a^	8.36 ± 0.16^ab^	46.34 ± 0.80^de^	41.27 ± 1.25^a^	67.00 ± 1.41^bcd^	2.32 ± 0.02^de^	39.03 ± 3.90^fg^
No verjuice, 72 h	5.67 ± 0.00^c^	–	40.49 ± 0.08^a^	19.19 ± 0.19^a^	8.42 ± 0.00^ab^	41.45 ± 0.45^h^	33.15 ± 4.78^a^	61.00 ± 1.41^efg^	2.81 ± 0.10^abc^	68.51 ± 2.78^abc^
30% verjuice, 72 h	4.47 ± 0.04^g^	10.89 ± 0.40^ab^	48.20 ± 0.01^a^	7.41 ± 0.09^a^	5.78 ± 0.07^b^	54.06 ± 0.49^a^	46.15 ± 1.46^a^	69.00 ± 1.41^abc^	2.87 ± 0.00^ab^	66.88 ± 11.44^bcd^
70% verjuice, 72 h	4.14 ± 0.02^h^	13.32 ± 0.42^a^	50.98 ± 0.57^a^	7.40 ± 0.01^a^	10.22 ± 0.12^ab^	49.48 ± 0.51^b^	40.76 ± 1.58^a^	71.00 ± 1.41^ab^	2.90 ± 0.04^ab^	44.03 ± 2.72^f^
100% verjuice, 72 h	3.66 ± 0.15^i^	14.74 ± 0.02^a^	55.55 ± 0.57^a^	7.27 ± 0.08^a^	14.85 ± 0.11^a^	47.46 ± 0.28^ed^	39.18 ± 0.01^a^	74.00 ± 0.00^a^	2.99 ± 0.13^a^	31.97 ± 2.74^g^

Abbreviations: MFI, myofibrillar fragmentation index; WBSF: Warner–Bratzler shear force; WHC: water‐holding capacity.

^1^ Values are mean ± standard deviation in two replicates.

#### 3.1.1 pH

As can be seen in Tables 2 and 3, pH was significantly affected by two variables of verjuice concentration (Table 2a) and marination time (Table 2b) as well as their interaction effects (Table 3). Sample's pH decreased as the marination time and verjuice concentration increased (*p* < .05) (Table 2a,b). The diffusion of verjuice acidic solution and salt into the meat texture lowers the pH of marinated samples. The rapid decline of pH over time is due probably to the leakage of lactate and meat proteins and the weakness of meat's buffering capacity in the pH range of 4.5 to 6 (Goli et al., 2014). In Table 2a, the first column assigned to the verjuice concentration of 0% includes samples only subjected to aging. Lactic acid produced after the cattle slaughter as a result of oxidation pathway cessation is the main reason for the decreased pH (from 6.84 to 5.89) for the samples during aging. Considering the fact that the presence of 2% salt in the acidic marinade diminishes protein isoelectric point (IP) approximately one unit (Hamm, 1975; Hudson, 1992), the sample treated with 100% verjuice solution for 48 h only showed a single unit drop among other treatments indicating that the sample pH is exactly at proteins' IP.

#### 3.1.2 Marinade uptake

Marinade uptake leading to weight gain and juiciness showed an increase (*p* < .05) upon exposure to verjuice solution (Table 2a); however, this increase was not significant with increasing verjuice concentration from 30% to 100% (*p* > .05). Among two mechanisms proposed by Goli et al. (2014), the electrostatic repulsions between proteins owing to the increase in positively charged groups following pH loss are unlikely cause for the increased mass gain after verjuice addition since the sample's pH is still above or close to proteins IP at most treatments. But protein hydration due to depolymerization and solubilization of myofibrillar proteins and connective tissue followed by structural weakening and inducing an open structure due to the interaction of the Cl^−^ ions with protein molecules at pH values above IP are more possible reasons for marinade uptake upon acidification (Tribuzi et al., 2014). Moreover, the swelling of filament lattice can be caused by cloud formed by sodium ions around filaments increasing the osmotic pressure within myofibrils (Cheng & Sun, 2008). The similar concentration of salt in all marination treatments and insignificant difference of mass gain for all three concentrations of verjuice marinade indicate more noticeable role of salt compared to acid in the water absorption of BF samples. As can be seen in Table 2b, marination time is a more efficient factor in marinade uptake than verjuice concentration, so that more prolonged marination time was accompanied by much more penetration of marinade into the meat texture. Interestingly, a deviation from the regular increasing trend of matter gain was observed with increasing acid concentration in the vicinity of proteins IP (100% verjuice, 48 h) (Table 3).

#### 3.1.3 Water‐holding capacity (WHC)

The comparison between the WHC of untreated BF samples (35.25%; Table 1) and that of samples stored without marination (42.35%; Table 2a) indicates that meat aging raises their WHC. Similarly, meat acidification increased WHC values by marinating BF blocks into the 30% verjuice solution (Table 2a). Since pH in the marinated samples is approaching isoelectric point with increasing acid concentration from 30% to 100%, a significant loss was observed for WHC. Kim et al. (2013) also found a slight decline in WHC with increasing soy sauce in marinade. However, marination time was only effective for the longest duration (Table 2b). For this reason, an increasing trend was only observed for samples kept until 72 h with increasing verjuice concentration from 30% to 100% (Table 3). The increased water holding observed for the treatment of 72 h can be probably ascribed to the pH drop below proteins' IP which causes swelling of fibers. In addition, pH in the 72‐h treatment was in favor of collagen swelling surrounding bundles of muscle fibers and single fibers (Cheng & Sun, 2008). Moreover, the proteolytic degradation of myofibrillar proteins by cathepsins B and D released from lysosome under acidic conditions can also trigger swelling of myofibrils and subsequently myo‐water retention (Hinikle, 2010; Straadt et al., 2007). Most importantly, enzymatic dissociation of peptide bonds may supply a number of polar groups to be bound to further water molecules. Nevertheless, this trend was opposite for other time intervals (Table 3) because pH was getting closer to the proteins' IP (Table 3). It is worth mentioning that greater amount of acid and salt gain in comparison with that of protein loss during marination could be the possible reason for incompatibility of the WHC results with those of marinade uptake as a function of acid concentration.

#### 3.1.4 Color measurements

L* and a* parameters are directly associated with meat color but the b* parameter is not directly related to meat color (Mancini & Hunt, 2005). The results in Table 3 exhibit that there is no significant interaction effect (*p* > .05) on the brightness of marinated and unmarinated BF samples. Furthermore, the brightness (L*) values were not affected by the marination time (Table 2b) (*p* > .05) but immersing BF samples in verjuice marinade regardless of the acid concentration significantly increased the L* values (Table 2a). Lightness consistency with increasing acid concentration can be explained due to the simultaneous incidence of, on the one hand, light reflection by approaching the proteins' IP increasing lightness (Hinikle, 2010), on the other hand, WHC reduction diminishing sample brightness.

According to Table 3, the interaction effects of marination time and verjuice concentration on a* index were just significant for BF samples immersed in the verjuice marinade. Like L* index, marinating meat with a combination of salt and verjuice, regardless of its concentration, had a reducing effect on a* values (*p* < .05). Raising marination time and verjuice concentration had no effect on the redness of meat samples (Table 2). The application of acetic, citric, and lactic acids in beef had similar effects on L* and a* values compared to the control (Arganosa & Marriott, 1989).

Myoglobin is the pigment that imparts color to meat, a color that relies upon the myoglobin oxidation state on the surface. The three myoglobin forms are as a result of their distinctive reflectance spectra. In meat surface, these three states are in a dynamic equilibrium driven by the oxygen presence and a number of other biochemical agents. In general, meat acidification and the low pressure of oxygen during immersion enhance the conversion of Mb to MetMb having a lower color intensity (Aktaş & Kaya, 2001; Mancini & Hunt, 2005). The results presented in Table 4 show that verjuice concentration is an effective factor in reducing total Mb content. The decreasing trend of MbO_2_ was the opposite of deoxyMb as a function of verjuice concentration. MetMb showed a nonsignificant increase with acid concentration (*p* > .05). The pH loss of muscle resulting from acid promotes pigment oxidation (Pohlman et al., 2002). Additionally, salt addition is accompanied by the growth of MetMb levels followed by lowering the MbO_2_ in muscles, because sodium chloride is able to delay activity of reductase enzymes in raw meat (Walczycka et al., 2005). Thus, interactive effects of salt and acid are responsible for the final color in muscles.

**TABLE 4:** (a) The main effects and (b) the interaction effects of verjuice concentration and marination time on total myoglobin content and percentage of deoxymyoglobin, oxymyoglobin, and metmyoglobin in *biceps femoris* beef muscles.

(a)				
a. Properties of treated BF^1^	Verjuice concentration %			
	0	30	70	100
Total myoglobin (mmol/L)	6.51 ± 1.79^a^	2.65 ± 0.19^b^	0.76 ± 0.00^b^	2.35 ± 0.09^b^
Deoxy Mb %	16.99 ± 2.77^b^	30.45 ± 3.94^ab^	22.96 ± 5.08^ab^	34.78 ± 3.07^a^
MbO_2_%	36.75 ± 1.21^a^	19.71 ± 1.95^ab^	24.86 ± 1.86^ab^	10.86 ± 1.55^b^
Met Mb %	46.61 ± 1.62^a^	55.08 ± 5.07^a^	52.26 ± 2.11^a^	60.80 ± 1.17^a^

b. Properties of treated BF	Marination time (h)			
	12	24	48	72
Total myoglobin (mmol/L)	3.87 ± 0.98^a^	2.40 ± 0.07^a^	2.25 ± 0.04^a^	2.75 ± 0.46^a^
Deoxy Mb %	24.32 ± 2.87^a^	30.44 ± 3.51^a^	31.68 ± 1.88^a^	23.74 ± 1.45^a^
MbO_2_%	23.20 ± 3.22^a^	21.49 ± 2.11^a^	17.40 ± 1.91^a^	18.86 ± 1.45^a^
Met Mb %	52.89 ± 6.81^a^	55.74 ± 5.62^a^	59.90 ± 3.45^a^	58.22 ± 3.13^a^

(b)				
Sample^1^				
	Total myoglobin (mmol/L)	Deoxy Mb %	MbO2%	Met Mb %
No verjuice, 12 h	8.55 ± 2.79^a^	15.77 ± 2.78^a^	42.32 ± 1.21^a^	42.17 ± 1.63^a^
30% verjuice, 12 h	5.26 ± 1.86^abcd^	24.93 ± 3.94^a^	16.14 ± 6.46^a^	59.72 ± 6.84^a^
70% verjuice, 12 h	2.04 ± 0.95^cd^	20.14 ± 5.08^a^	23.36 ± 4.99^a^	57.20 ± 9.96^a^
100% verjuice, 12 h	0.39 ± 0.00^d^	36.45 ± 6.79^a^	10.97 ± 0.72^a^	52.45 ± 3.75^a^
No verjuice, 24 h	7.76 ± 1.67^ab^	19.57 ± 3.07^a^	30.53 ± 8.15^a^	50.46 ± 5.07^a^
30% verjuice, 24 h	2.83 ± 1.39^bcd^	18.23 ± 2.36^a^	27.59 ± 0.68^a^	53.84 ± 3.24^a^
70% verjuice, 24 h	0.39 ± 0.19^d^	40.98 ± 6.92^a^	4.95 ± 0.26^a^	53.77 ± 5.62^a^
100% verjuice, 24 h	0.20 ± 0.09^d^	45.00 ± 3.03^a^	2.32 ± 1.73^a^	52.90 ± 8.92^a^
No Verjuice, 48 h	6.18 ± 0.19^abc^	15.49 ± 1.88^a^	37.41 ± 3.81^a^	46.29 ± 2.11^a^
30% verjuice, 48 h	1.78 ± 0.95^cd^	30.57 ± 8.74^a^	15.09 ± 5.52^a^	55.76 ± 4.62^a^
70% verjuice, 48 h	0.39 ± 0.17^d^	26.89 ± 4.86^a^	21.10 ± 10.02^a^	52.24 ± 3.45^a^
100% verjuice, 48 h	0.20 ± 0.00^d^	48.78 ± 0.32^a^	1.11 ± 3.31^a^	62.33 ± 5.99^a^
No verjuice, 72 h	6.58 ± 0.75^abc^	17.13 ± 0.51^a^	35.75 ± 1.95^a^	47.52 ± 1.17^a^
30% verjuice, 72 h	0.72 ± 0.26^d^	29.08 ± 3.51^a^	21.87 ± 6.23^a^	49.01 ± 9.34^a^
70% verjuice, 72 h	0.20 ± 0.09^d^	31.83 ± 9.19^a^	23.05 ± 2.55^a^	45.82 ± 5.62^a^
100% verjuice, 72 h	0.15 ± 0.02^d^	24.91 ± 0.15^a^	10.88 ± 1.33^a^	64.54 ± 1.60^a^

^1^ Values are mean ± standard deviation in two replicates.

#### 3.1.5 Cooking loss

Table 3 shows that aging without marination increased cooking loss because of fibers disintegration during storage (Straadt et al., 2007) and greater cooking loss was observed following marination that might be caused by holes created by injection providing channels through which the marinade exits. More importantly, the breakdown and solubilization of collagen existing in endomysium and perimysium under acidic conditions create gaps between muscle fibers facilitating water diffuse‐out (Kim et al., 2013). Unexpectedly, although poor WHC leads to a high drip of meat during cooking (Hughes et al., 2014), our results in Table 2a showed that there is less cookout with increasing acid concentration in verjuice solutions which is parallel to decreasing WHC. It should be noted that the decrease in cooking loss was only significant (*p* < .05) for the highest concentration of acid. It can be concluded that water‐holding capacity cannot be only parameter effective on the cookout but also the ease of mass transfer process during cooking can contribute to diffusing out of water. In this study, the swelling ascribed to pH drop was marginal during marination, but the role of stromal proteins in swelling cannot be excluded and their impact was revealed during their heating. The swollen collagen can be readily converted into gelatin at high temperatures (Kim et al., 2013) and water diffusion may be hindered in the gelatinized connective tissue covering fibers. Hence, it seems there is a poor correlation between WHC and the water lost overcooking. Likewise, many studies also encountered increased water loss during cooking as the beef pH decreased (Aktaş & Kaya, 2001; Aktaş et al., 2003; Gault, 1984, 1985, 1991; Önenç et al., 2004). As shown in Table 2b, marination time had no pronounced effect on cooking loss. The salt concentrations higher than 1.5% by screening the positive charge groups of proteins at low pHs prevent from revealing the impact of pH loss during marinating on cookout (Goli et al., 2014). The interaction effect of time and concentration on drip loss during cooking was also insignificant (Table 3).

#### 3.1.6 Warner–Bratzler shear force (WBSF)

The results in Table 2a,b show that the effects of verjuice concentration and marination time were significant on WBSF reduction (*p* < .05). According to Table 3, pH loss below proteins' IP happened in the marination treatments for 72 h. Since a decreasing trend for Warner–Bratzler shear force was observed in all time treatments with increasing verjuice concentration (Table 3), the enhanced tenderness cannot be only attributed to the increased immobilized water content because Cl^−^ ions are able to neutralize the –NH_3_^+^ groups resulted from pH loss to some extent. Less toughness in meats soaked in acidic marinade with increasing both acid concentration and marination time can be related to the collagen solubilization under acidic conditions through peptide bond hydrolysis and breakdown of cross‐links. The pH and heat lability of aldimine bonds constituting up a part of the mentioned cross‐links as well as the increase in the rate of aspartyl peptide bond hydrolysis with pH loss from 6 to 4 are two proposed mechanisms for collagen dissolution (Offer & Knight, 1988). As mentioned earlier, another possibility is that the lowering of BF's pH in the marinade enhances the proteolytic activity of cathepsins whose optimal pH is in the range of 3.5 to 5 (Burke & Monahan, 2003). Thus, proteolytic weakening of muscle proteins can be in part responsible for the tenderness. Other studies also proved that meat marinating with acidic solutions is accompanied by reduced WBSF values (Aktaş et al., 2003; Istrati et al., 2015; Sengun et al., 2020). Considering 46 N as acceptable WBSF for commercial beef, the samples marinated with 70% and 100% verjuice achieved acceptable tenderness after 72 and 24 h, respectively (Table 3).

#### 3.1.7 Myofibrillar fragmentation index (MFI)

The meat tenderness is highly dependent on the weakening and breakdown of myofibrillar structures that was followed by MFI and SDS‐PAGE analyses. As can be seen in Table 2a,b, both acid concentration and marination time affect MFI significantly (*p* < .05) as it exhibited a growing tendency with increasing of both variables.

The intensity of BF tenderness based on MFI value is classified as tender (MFI > 60), intermediate (MFI = 50–60), and tough (MFI < 50) (Culler et al., 1978). The results show that the raw meat used in this study was totally tough with MFI of about 40 (Table 1) and the index varied from intermediate to tender by immersing in acidic marinade (Table 3). It is clear from Table 3 that meat acidification helps greater improvement of its MFI value which can be explained by the formation of low molecular weight peptides resulting from enzymatic proteolysis of muscle proteins (Kim et al., 2013).

Three types of proteinase including the calpain, the lysosomal cathepsins, and multi catalytic proteinase complex are involved in postmortem proteolysis of skeletal muscle. Although the high proteolytic activity of μ‐calpain (a calcium‐dependent natural protease) and cathepsin L plays an important role in postmortem proteolysis of myofibrils during aging, their activity would be restricted under acidic conditions during marination (Geesink et al., 2006; Kim et al., 2013). Additionally, the less activity of m‐calpain and μ‐calpain in BF than other muscles (Wheeler et al., 2000) makes the role of nonlysosomal protease peripheral in myofibrils fragmentation during marination. Myofibrillar protein degradation (especially the heavy chain of myosin, titin, M‐ and C‐proteins, tropomyosin and troponin‐T and ‐I) due to the activity of cathepsins, particularly cathepsin D which is more active in acidic pH range (pH 3–5) is a more probable reason for the increased MFI values (Goll et al., 1983; Hughes et al., 1999, 2000; Matsukura et al., 1981).

#### 3.1.8 SDS–PAGE

**FIGURE 1:**
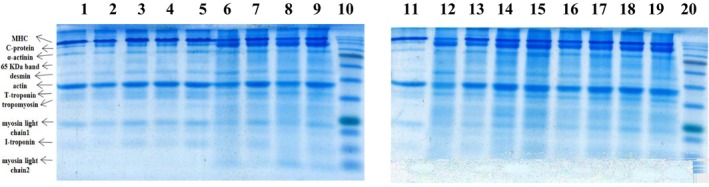
SDS–PAGE images of myofibrillar proteins from *biceps femoris* beef muscles marinated with various verjuice levels (0%, 30%, 70%, and 100%) at different times (12, 24, 48, and 72 h). (1) control; (2) no verjuice, 12 h; (3) no verjuice, 24 h; (4) no verjuice, 48 h; (5) no verjuice, 72 h; (6) 30% verjuice, 12 h; (7) 30% verjuice, 24 h; (8) 30% verjuice, 48 h; (9) 30% verjuice, 72 h; (10) marker; (11) control 2; (12) 70% verjuice, 12 h; (13) 70% verjuice, 24 h; (14) 70% verjuice, 48 h; (15) 70% verjuice, 72 h; (16) 100% verjuice, 12 h; (17) 100% verjuice, 24 h; (18) 100% verjuice, 48 h; (19) 100% verjuice, 72 h; (20) marker.

The effect of marination on the bands obtained by SDS–PAGE gels is depicted in Figure 1 in which high molecular weight proteins including myosin, C‐protein, desmin, α‐actinin, actin, tropomyosin, and troponin‐T are noticeable. According to the results in Table 5, although aging at 4 °C had no significant effect (*p* > .05) on the mean band area of myosin heavy chain (MHC), it pronouncedly decreased under the impact of acid concentration (*p* < .05). Thus, myosin degradation does not contribute to BF tenderness during cold storage. The decreased MHC densities for samples marinated with verjuice were coincident with the increased band intensities of C‐protein with a molecular weight of 140–145 kDa as well as α‐actinin with a molecular weight of 95 kDa in comparison with unmarinated samples. The presence of peptides with molecular weight similar to C‐protein is more obvious than those having molecular weight equivalent to α‐actinin. This fact is in line with findings obtained by other researchers that cathepsin breaks down MHC into peptides of 50–95, 150, and 160–180 kDa over time (Bandman & Zdanis, 1988; Goll et al., 1983; Sentandreu et al., 2002; Yates et al., 1983). Meat acidification through the release of cathepsins into cytoplasm following lysosome membrane rupture as well as increasing cathepsin activity plays an important role in meat tenderization (Kemp et al., 2010; Sentandreu et al., 2002; Taylor et al., 1995). The constancy of band ascribed to actin (MW = 43 kDa) during storage alone (*p* > .05) indicates that its proteolysis does not occur during aging. Interestingly, the incremental trend (*p* < .05) observed in the band area of actin after acidic marinating indicates that catheptic degradation occurs only for myosin leading to intensified actin band. The susceptibility of MHC to proteolysis by cathepsin D, B, or L has been already confirmed (Schwartz & Bird, 1977).

**TABLE 5:** Changes of protein band intensity in 12.5% SDS–PAGE gel from *biceps femoris* beef muscles marinated with various verjuice levels (0%, 30%, 70%, and 100%) in different times (12, 24, 48, and 72 h).

Band/protein	Samples	Molecular weight	R_f_	Band area
Band 1 (Myosin Heavy Chain)	No verjuice, 12 h	214.710 ± 5.247^a^	0.096 ± 0.020^a^	6680 ± 339.411^ab^
	30% verjuice, 12 h	209.921 ± 2.230^ab^	0.098 ± 0.026^a^	6641 ± 360.624^a^
	70% verjuice, 12 h	210.328 ± 0.320^ab^	0.097 ± 0.000^a^	6078 ± 79.903^abcde^
	100% verjuice, 12 h	213.304 ± 3.740^ef^	0.091 ± 0.001^a^	5778 ± 82.024^cdef^
	No verjuice, 24 h	215.890 ± 0.686^a^	0.089 ± 0.013^a^	6366 ± 104.652^abc^
	30% verjuice, 24 h	217.798 ± 1.483^a^	0.087 ± 0.007^a^	6158 ± 70.711^abcd^
	70% verjuice, 24 h	213.960 ± 2.336^bcd^	0.091 ± 0.004^a^	5862 ± 110.309^bcdef^
	100% verjuice, 24 h	211.190 ± 2.335^def^	0.092 ± 0.005^a^	5513 ± 168.291^def^
	No Verjuice, 48 h	212.046 ± 2.970^ab^	0.099 ± 0.022^a^	6320 ± 1136.137^abc^
	30% verjuice, 48 h	210.197 ± 2.402^ab^	0.099 ± 0.026^a^	6088 ± 141.421^abcde^
	70% verjuice, 48 h	211.664 ± 1.523^ef^	0.095 ± 0.006^a^	5476 ± 62.225^ef^
	100% verjuice, 48 h	208.856 ± 3.149^fg^	0.099 ± 0.004^a^	540.9 ± 86.267^f^
	No verjuice, 72 h	210.084 ± 1.416^ab^	0.096 ± 0.026^a^	6086 ± 82.024^abcde^
	30% verjuice, 72 h	207.638 ± 2.044^abc^	0.099 ± 0.023^a^	5930 ± 161.220^bcdef^
	70% verjuice, 72 h	208.458 ± 3.204^cde^	0.098 ± 0.001^a^	5855 ± 125.865^bcdef^
	100% verjuice, 72 h	209.834 ± 0.462^g^	0.097 ± 0.001^a^	5339 ± 176.777^f^
	No verjuice, 12 h	139.730 ± 1.540^f^	0.071 ± 0.001^a^	4016 ± 90.510^a^
	30% verjuice, 12 h	140.285 ± 1.276^f^	0.070 ± 0.001^ab^	4157 ± 18.385^a^
	70% verjuice, 12 h	142.848 ± 0.421^bcdef^	0.068 ± 0.001^abcd^	4203 ± 94.752^a^
	100% verjuice, 12 h	142.856 ± 0.592^bcdef^	0.068 ± 0.002^abcd^	4269 ± 69.296^a^
	No verjuice, 24 h	141.139 ± 0.399^ef^	0.066 ± 0.001^abc^	5017 ± 1304.612^a^
	30% verjuice, 24 h	140.612 ± 0.560^f^	0.067 ± 0.001^ab^	4126 ± 7.778^a^
	70% verjuice, 24 h	145.584 ± 0.815^abc^	0.066 ± 0.001^bcd^	4370 ± 22.627^a^
Band 2 (C‐protein)	100% verjuice, 24 h	145.928 ± 0.802^ab^	0.068 ± 0.001^abcd^	4305 ± 29.698^a^
	No Verjuice, 48 h	142.359 ± 0.727^cdef^	0.067 ± 0.003^abcd^	4108 ± 45.255^a^
	30% verjuice, 48 h	142.161 ± 0.888^def^	0.069 ± 0.003^abcd^	4152 ± 45.962^a^
	70% verjuice, 48 h	144.122 ± 0.339^abcde^	0.067 ± 0.001^abcd^	4267 ± 40.305^a^
	100% verjuice, 48 h	147.367 ± 1.289^a^	0.065 ± 0.001^cd^	4423 ± 40.305^a^
	No verjuice, 72 h	144.706 ± 1.114^abcd^	0.066 ± 0.001^bcd^	4339 ± 79.903^a^
	30% verjuice, 72 h	142.411 ± 0.614^cdef^	0.068 ± 0.001^abcd^	4188 ± 28.284^a^
	70% verjuice, 72 h	144.814 ± 0.263^abcd^	0.067 ± 0.001^abcd^	4393 ± 6.364^a^
	100% verjuice, 72 h	146.963 ± 0.419^a^	0.068 ± 0.000^d^	4546 ± 65.054^a^
	No verjuice, 12 h	95.906 ± 1.037^c^	0.164 ± 0.005^a^	4116 ± 31.113^f^
	30% verjuice, 12 h	96.430 ± 0.759^c^	0.160 ± 0.002^ab^	4334 ± 87.681^cdef^
	70% verjuice, 12 h	98.722 ± 0.026^bc^	0.158 ± 0.000^ab^	4378 ± 36.770^cdef^
	100% verjuice, 12 h	98.917 ± 0.107^bc^	0.157 ± 0.001^ab^	4648 ± 101.823^cd^
	No verjuice, 24 h	95.650 ± 0.248^c^	0.164 ± 0.001^a^	4206 ± 59.397^ef^
	30% verjuice, 24 h	98.830 ± 1.655^bc^	0.161 ± 0.004^ab^	4323 ± 37.477^cdef^
	70% verjuice, 24 h	101.596 ± 3.229^abc^	0.157 ± 0.004^ab^	4602 ± 115.966^cde^
Band 3 (α‐actinin)	100% verjuice, 24 h	98.746 ± 1.773^bc^	0.164 ± 0.001^a^	4332 ± 96.167^cdef^
	No Verjuice, 48 h	96.281 ± 0.144^c^	0.163 ± 0.000^a^	4260 ± 28.284^def^
	30% verjuice, 48 h	96.528 ± 2.161^c^	0.162 ± 0.002^ab^	4327 ± 86.267^cdef^
	70% verjuice, 48 h	101.916 ± 3.176^abc^	0.158 ± 0.002^ab^	4724 ± 271.529^bc^
	100% verjuice, 48 h	104.508 ± 1.901^ab^	0.156 ± 0.003^ab^	5091 ± 97.581^ab^
	No verjuice, 72 h	98.263 ± 0.624^bc^	0.162 ± 0.004^ab^	4450 ± 132.936^cdef^
	30% verjuice, 72 h	98.790 ± 0.400^bc^	0.159 ± 0.001^ab^	4600 ± 59.569^cde^
	70% verjuice, 72 h	103.963 ± 0.164^ab^	0.158 ± 0.001^ab^	5153 ± 18.385^a^
	100% verjuice, 72 h	106.588 ± 1.503^a^	0.153 ± 0.001^b^	5256 ± 23.355^a^
	No verjuice, 12 h	64.548 ± 0.324^de^	0.246 ± 0.002^bcd^	4802 ± 82.024^f^
	30% verjuice, 12 h	65.626 ± 0.135^abc^	0.240 ± 0.001^cdef^	5302 ± 113.137^def^
	70% verjuice, 12 h	65.836 ± 0.166^bcde^	0.238 ± 0.000^bc^	5152 ± 19.799^def^
	100% verjuice, 12 h	64.908 ± 1.129^bcde^	0.247 ± 0.001^def^	5267 ± 64.347^def^
	No verjuice, 24 h	64.743 ± 0.509^cde^	0.246 ± 0.001^ef^	5431 ± 365.574^de^
	30% verjuice, 24 h	65.604 ± 1.257^abcd^	0.241 ± 0.001^cdef^	5638 ± 180.312^cd^
	70% verjuice, 24 h	66.203 ± 0.132^a^	0.238 ± 0.001^cdef^	6077 ± 64.347^bc^
Band 4 (65 kDa)	100% verjuice, 24 h	64.721 ± 1.148^cde^	0.247 ± 0.002^b^	5135 ± 97.58^def^
	No Verjuice, 48 h	65.833 ± 0.074^ab^	0.240 ± 0.002^f^	6462 ± 229.103^b^
	30% verjuice, 48 h	64.684 ± 0.055^cde^	0.248 ± 0.001^bcdef^	5509 ± 53.033^cde^
	70% verjuice, 48 h	65.461 ± 0.109^abcd^	0.241 ± 0.002^bcde^	5650 ± 74.246^cd^
	100% verjuice, 48 h	64.257 ± 0.258^e^	0.247 ± 0.001^bcde^	5278 ± 234.052^def^
	No verjuice, 72 h	66.327 ± 0.005^a^	0.238 ± 0.000^bcdef^	7173 ± 63.640^a^
	30% verjuice, 72 h	65.542 ± 0.492^abcd^	0.243 ± 0.001^bcdef^	5609 ± 78.489^cd^
	70% verjuice, 72 h	64.710 ± 0.151^cde^	0.247 ± 0.001^bcd^	5314 ± 26.163^def^
	100% verjuice, 72 h	62.870 ± 0.508^f^	0.250 ± 0.001^a^	5018 ± 45.255^ef^
	No verjuice, 12 h	49.637 ± 0.393^a^	0.390 ± 0.002^a^	11,056 ± 927.724^a^
	30% verjuice, 12 h	49.042 ± 0.319^abc^	0.394 ± 0.001^a^	11,514 ± 1264.307^a^
	70% verjuice, 12 h	48.668 ± 0.322^abc^	0.394 ± 0.001^a^	11,606 ± 398.808^a^
	100% verjuice, 12 h	47.787 ± 0.568^abc^	0.396 ± 0.001^a^	11,465 ± 1845.549^a^
	No verjuice, 24 h	48.860 ± 0.318^abc^	0.393 ± 0.000^a^	10,620 ± 0.000^a^
	30% verjuice, 24 h	48.339 ± 0.480^abc^	0.394 ± 0.001^a^	10,648 ± 893.783^a^
	70% verjuice, 24 h	48.929 ± 0.860^abc^	0.393 ± 0.004^a^	11,038 ± 534.573^a^
Band 5 (Desmin)	100% verjuice, 24 h	48.440 ± 0.778^abc^	0.394 ± 0.004^a^	10,385 ± 597.505^a^
	No Verjuice, 48 h	47.639 ± 0.053^bc^	0.393 ± 0.001^a^	9961 ± 219.203^a^
	30% verjuice, 48 h	48.760 ± 1.032^abc^	0.392 ± 0.013^a^	10,152 ± 268.701^a^
	70% verjuice, 48 h	49.447 ± 0.433^ab^	0.388 ± 0.001^a^	10,825 ± 255.266^a^
	100% verjuice, 48 h	49.311 ± 0.472^ab^	0.390 ± 0.008^a^	10,648 ± 304.763^a^
	No verjuice, 72 h	47.119 ± 0.136^c^	0.391 ± 0.001^a^	9780 ± 73.539^a^
	30% verjuice, 72 h	48.924 ± 0.227^abc^	0.392 ± 0.001^a^	11,157 ± 185.262^a^
	70% verjuice, 72 h	48.826 ± 0.143^abc^	0.391 ± 0.000^a^	11,199 ± 271.529^a^
	100% verjuice, 72 h	49.136 ± 0.156^ab^	0.392 ± 0.002^a^	11,203 ± 332.340^a^
	No verjuice, 12 h	44.606 ± 0.520^a^	0.398 ± 0.011^ab^	8208 ± 124.451^b^
	30% verjuice, 12 h	43.731 ± 0.232^a^	0.398 ± 0.001^b^	7897 ± 98.288^b^
	70% verjuice, 12 h	44.622 ± 0.157^a^	0.395 ± 0.001^ab^	8198 ± 217.789^b^
	100% verjuice, 12 h	42.866 ± 1.057^a^	0.403 ± 0.008^ab^	7840 ± 118.794^b^
	No verjuice, 24 h	44.584 ± 0.155^a^	0.397 ± 0.003^ab^	8168 ± 47.376^b^
	30% verjuice, 24 h	43.718 ± 0.346^a^	0.399 ± 0.009^ab^	7865 ± 52.326^b^
	70% verjuice, 24 h	44.118 ± 0.581^a^	0.395 ± 0.001^ab^	8202 ± 109.602^b^
Band 6 (Actin)	100% verjuice, 24 h	43.707 ± 0.977^a^	0.401 ± 0.008^ab^	8778 ± 110.309^b^
	No Verjuice, 48 h	44.731 ± 0.071^a^	0.397 ± 0.003^ab^	8175 ± 32.527^b^
	30% verjuice, 48 h	42.656 ± 0.260^a^	0.406 ± 0.003^a^	7645 ± 72.125^b^
	70% verjuice, 48 h	43.788 ± 1.049^a^	0.402 ± 0.008^ab^	8125 ± 222.032^b^
	100% verjuice, 48 h	43.106 ± 1.114^a^	0.402 ± 0.008^ab^	8619 ± 216.375^b^
	No verjuice, 72 h	44.418 ± 0.252^a^	0.397 ± 0.004^ab^	8143 ± 8.485^b^
	30% verjuice, 72 h	43.052 ± 1.091^a^	0.403 ± 0.011^ab^	7584 ± 79.196^b^
	70% verjuice, 72 h	43.325 ± 0.820^a^	0.404 ± 0.006^ab^	10,848 ± 873.984^a^
	100% verjuice, 72 h	43.152 ± 0.018^a^	0.400 ± 0.001^ab^	11,395 ± 1025.305^a^
	No verjuice, 12 h	39.572 ± 0.632^a^	0.455 ± 0.014^a^	6324 ± 50.912^a^
	30% verjuice, 12 h	39.438 ± 0.377^abcd^	0.448 ± 0.001^a^	5844 ± 28.284^ab^
	70% verjuice, 12 h	38.144 ± 0.644^abcde^	0.457 ± 0.008^a^	5504 ± 330.926^bc^
	100% verjuice, 12 h	37.006 ± 1.074^cde^	0.466 ± 0.008^a^	5342 ± 144.250^bc^
	No verjuice, 24 h	39.312 ± 0.071^ab^	0.457 ± 0.019^a^	5956 ± 79.196^ab^
	30% verjuice, 24 h	38.194 ± 0.095^abcde^	0.453 ± 0.001^a^	5675 ± 230.517^ab^
	70% verjuice, 24 h	37.428 ± 0.539^bcde^	0.462 ± 0.001^a^	5822 ± 166.877^ab^
Band 7 (Troponin‐T)	100% verjuice, 24 h	36.582 ± 0.060^de^	0.469 ± 0.001^a^	5714 ± 285.671^ab^
	No Verjuice, 48 h	38.860 ± 0.023^abc^	0.455 ± 0.029^a^	5652 ± 73.539^ab^
	30% verjuice, 48 h	37.593 ± 0.467^bcde^	0.458 ± 0.002^a^	5542 ± 353.740^abc^
	70% verjuice, 48 h	36.998 ± 0.731^cde^	0.466 ± 0.005^a^	5347 ± 189.505^bc^
	100% verjuice, 48 h	36.441 ± 0.452^e^	0.470 ± 0.004^a^	5255 ± 352.139^bc^
	No verjuice, 72 h	37.444 ± 0.355^bcde^	0.462 ± 0.002^a^	5557 ± 318.198^abc^
	30% verjuice, 72 h	37.950 ± 0.095^abcde^	0.450 ± 0.011^a^	5528 ± 237.588^abc^
	70% verjuice, 72 h	37.368 ± 0.124^cde^	0.460 ± 0.001^a^	5146 ± 84.853^bc^
	100% verjuice, 72 h	37.294 ± 0.226^cde^	0.464 ± 0.003^a^	4759 ± 145.664^c^
	No verjuice, 12 h	36.060 ± 0.653^a^	0.483 ± 0.013^a^	6156 ± 786.303^a^
	30% verjuice, 12 h	35.792 ± 0.559^a^	0.488 ± 0.013^a^	6241 ± 77.782^a^
	70% verjuice, 12 h	35.712 ± 0.398^a^	0.486 ± 0.000^a^	6051 ± 123.037^a^
	100% verjuice, 12 h	35.416 ± 0.452^a^	0.490 ± 0.001^a^	6228 ± 152.735^a^
	No verjuice, 24 h	35.862 ± 0.754^a^	0.484 ± 0.012^a^	6138 ± 172.534^a^
	30% verjuice, 24 h	35.616 ± 0.539^a^	0.496 ± 0.001^a^	6081 ± 111.723^a^
	70% verjuice, 24 h	35.550 ± 0.455^a^	0.497 ± 0.001^a^	6002 ± 53.740^a^
Band 8 (Tropomyosin)	100% verjuice, 24 h	34.792 ± 0.006^a^	0.500 ± 0.003^a^	5948 ± 22.627^a^
	No Verjuice, 48 h	34.510 ± 0.612^a^	0.498 ± 0.001^a^	6031 ± 156.978^a^
	30% verjuice, 48 h	34.116 ± 0.091^a^	0.502 ± 0.006^a^	5934 ± 31.113^a^
	70% verjuice, 48 h	35.286 ± 0.354^a^	0.492 ± 0.001^a^	5993 ± 12.728^a^
	100% verjuice, 48 h	35.047 ± 0.987^a^	0.492 ± 0.006^a^	5881 ± 77.782^a^
	No verjuice, 72 h	34.550 ± 0.422^a^	0.494 ± 0.002^a^	6184 ± 214.960^a^
	30% verjuice, 72 h	34.226 ± 0.060^a^	0.494 ± 0.001^a^	6002 ± 19.799^a^
	70% verjuice, 72 h	34.798 ± 0.274^a^	0.490 ± 0.002^a^	5928 ± 8.485^a^
	100% verjuice, 72 h	34.490 ± 0.257^a^	0.493 ± 0.001^a^	5972 ± 69.286^a^
	No verjuice, 12 h	–	–	–
	30% verjuice, 12 h	29.254 ± 0.067^a^	0.584 ± 0.001^ab^	9981 ± 72.125^d^
	70% verjuice, 12 h	29.546 ± 0.510^a^	0.579 ± 0.006^ab^	10,398 ± 828.729^d^
	100% verjuice, 12 h	30.022 ± 0.162^a^	0.562 ± 0.018^b^	11,048 ± 393.151^cd^
	No verjuice, 24 h	–	–	–
	30% verjuice, 24 h	29.702 ± 0.233^a^	0.577 ± 0.004^ab^	12,267 ± 804.688^bcd^
	70% verjuice, 24 h	30.225 ± 0.056^a^	0.569 ± 0.001^ab^	12,276 ± 1396.496^bcd^
Band 9 (30 kDa)	100% verjuice, 24 h	30.300 ± 0.095^a^	0.570 ± 0.001^ab^	14,017 ± 1243.094^ab^
	No Verjuice, 48 h	–	–	–
	30% verjuice, 48 h	29.597 ± 0.508^a^	0.582 ± 0.004^ab^	13,012 ± 328.098^abcd^
	70% verjuice, 48 h	29.825 ± 0.781^a^	0.570 ± 0.004^ab^	15,304 ± 1001.263^abc^
	100% verjuice, 48 h	30.496 ± 0.087^a^	0.559 ± 0.010^b^	17,045 ± 948.937^a^
	No verjuice, 72 h	–	–	–
	30% verjuice, 72 h	30.282 ± 0.420^a^	0.597 ± 0.002^a^	13,878 ± 443.356^abcd^
	70% verjuice, 72 h	30.435 ± 0.501^a^	0.560 ± 0.017^b^	16,446 ± 1951.615^ab^
	100% verjuice, 72 h	29.420 ± 0.440^a^	0.576 ± 0.001^ab^	16,535 ± 612.354^ab^
	No verjuice, 12 h	24.594 ± 00638^a^	0.666 ± 0.004^a^	6164 ± 107.480^f^
	30% verjuice, 12 h	25.266 ± 0.066^a^	0.660 ± 0.002^a^	6516 ± 28.284^f^
	70% verjuice, 12 h	26.480 ± 0.588^a^	0.660 ± 0.013^a^	8104 ± 113.137^abc^
	100% verjuice, 12 h	26.574 ± 0.028^a^	0.652 ± 0.004^a^	8291 ± 26.870^abc^
	No verjuice, 24 h	24.694 ± 0.432^a^	0.674 ± 0.006^a^	6564 ± 220.617^f^
	30% verjuice, 24 h	25.085 ± 0.703^a^	0.658 ± 0.004^a^	6807 ± 97.581^ef^
	70% verjuice, 24 h	25.344 ± 0.116^a^	0.665 ± 0.003^a^	7824 ± 135.765^bcd^
Band 10 (Myosin Light Chain1)	100% verjuice, 24 h	26.368 ± 0.521^a^	0.654 ± 0.009^a^	6596 ± 469.519^f^
	No Verjuice, 48 h	24.857 ± 0.662^a^	0.670 ± 0.008^a^	7096 ± 22.627^def^
	30% verjuice, 48 h	26.001 ± 0.130^a^	0.666 ± 0.001^a^	7685 ± 210.718^bcde^
	70% verjuice, 48 h	25.224 ± 0.976^a^	0.657 ± 0.009^a^	8920 ± 280.014^a^
	100% verjuice, 48 h	26.068 ± 1.019^a^	0.653 ± 0.007^a^	7705 ± 436.992^bcde^
	No verjuice, 72 h	25.312 ± 0.825^a^	0.658 ± 0.006^a^	7561 ± 227.688^cde^
	30% verjuice, 72 h	25.628 ± 0.474^a^	0.668 ± 0.008^a^	8032 ± 169.706^abcd^
	70% verjuice, 72 h	25.771 ± 0.872^a^	0.664 ± 0.001^a^	7726 ± 141.421^bcde^
	100% verjuice, 72 h	26.410 ± 0.284^a^	0.656 ± 0.004^a^	8620 ± 393.151^ab^
	No verjuice, 12 h	22.214 ± 0.285^ab^	0.688 ± 0.005^abc^	7426 ± 65.054^cd^
	30% verjuice, 12 h	22.415 ± 0.034^ab^	0.688 ± 0.001^abc^	7768 ± 84.853^bc^
	70% verjuice, 12 h	22.881 ± 0.229^a^	0.681 ± 0.004^abc^	8368 ± 130.108^abc^
	100% verjuice, 12 h	22.459 ± 0.431^ab^	0.679 ± 0.006^bc^	7980 ± 254.558^abc^
	No verjuice, 24 h	21.824 ± 0.059^ab^	0.695 ± 0.001^abc^	7970 ± 70.711^abc^
	30% verjuice, 24 h	22.532 ± 0.305^a^	0.699 ± 0.001^ab^	8213 ± 12.278^abc^
	70% verjuice, 24 h	22.569 ± 0.275^a^	0.669 ± 0.002^c^	7781 ± 57.983^bc^
Band 11 (Troponin‐I)	100% verjuice, 24 h	20.350 ± 0.366^c^	0.705 ± 0.001^a^	8280 ± 101.823^abc^
	No Verjuice, 48 h	22.008 ± 0.141^ab^	0.701 ± 0.009^ab^	6467 ± 527.502^d^
	30% verjuice, 48 h	22.066 ± 0.081^ab^	0.706 ± 0.003^a^	8589 ± 502.046^abc^
	70% verjuice, 48 h	22.586 ± 0.384^a^	0.703 ± 0.011^ab^	8983 ± 465.276^ab^
	100% verjuice, 48 h	22.547 ± 0.291^a^	0.701 ± 0.006^ab^	9195 ± 354.968^a^
	No verjuice, 72 h	22.137 ± 0.052^ab^	0.700 ± 0.009^ab^	8910 ± 692.965^ab^
	30% verjuice, 72 h	22.725 ± 0.567^a^	0.684 ± 0.014^abc^	8866 ± 381.838^ab^
	70% verjuice, 72 h	21.216 ± 0.644^bc^	0.702 ± 0.001^ab^	9211 ± 26.870^a^
	100% verjuice, 72 h	21.780 ± 0.110^ab^	0.700 ± 0.004^ab^	8787 ± 142.836^ab^
	No verjuice, 12 h	17.934 ± 0.077^a^	0.810 ± 0.002^ab^	6540 ± 84.853^f^
	30% verjuice, 12 h	17.904 ± 0.248^a^	0.810 ± 0.001^ab^	11,693 ± 584.070^de^
	70% verjuice, 12 h	17.588 ± 0.268^a^	0.811 ± 0.011^ab^	14,206 ± 625.082^bcd^
	100% verjuice, 12 h	17.359 ± 0.313^a^	0.816 ± 0.002^ab^	13,643 ± 3186.223^bcd^
	No verjuice, 24 h	18.278 ± 0.283^a^	0.800 ± 0.002^ab^	8354 ± 913.582^ef^
	30% verjuice, 24 h	18.305 ± 0.447^a^	0.794 ± 0.015^b^	13,046 ± 1224.709^cde^
	70% verjuice, 24 h	17.625 ± 0.200^a^	0.816 ± 0.006^ab^	18,532 ± 774.989^ab^
Band 12 (Myosin Light Chain2)	100% verjuice, 24 h	17.084 ± 0.382^a^	0.827 ± 0.006^ab^	17,416 ± 1255.822^abc^
	No Verjuice, 48 h	18.152 ± 0.196^a^	0.803 ± 0.004^ab^	11,686 ± 1535.836^de^
	30% verjuice, 48 h	17.636 ± 0.477^a^	0.822 ± 0.018^ab^	16,818 ± 1473.611^abcd^
	70% verjuice, 48 h	17.174 ± 0.931^a^	0.820 ± 0.017^ab^	21,900 ± 243.245^a^
	100% verjuice, 48 h	17.352 ± 0.037^a^	0.833 ± 0.004^a^	12,577 ± 1002.677^cde^
	No verjuice, 72 h	18.313 ± 0.163^a^	0.794 ± 0.001^b^	11,783 ± 179.605^de^
	30% verjuice, 72 h	18.030 ± 0.324^a^	0.806 ± 0.007^ab^	17,524 ± 1804.537^abc^
	70% verjuice, 72 h	17.602 ± 0.155^a^	0.820 ± 0.007^ab^	13,528 ± 859.842^bcd^
	100% verjuice, 72 h	16.878 ± 0.292^a^	0.819 ± 0.009^a^	16,543 ± 1172.383^bcd^
	No verjuice, 12 h	–	–	–
	30% verjuice, 12 h	12.196 ± 0.368^a^	0.939 ± 0.005^a^	9071 ± 731.148^a^
	70% verjuice, 12 h	10.829 ± 0.496^a^	0.946 ± 0.001^a^	9480 ± 780.646^a^
	100% verjuice, 12 h	10.484 ± 0.308^a^	0.952 ± 0.004^a^	10,908 ± 1001.263^a^
	No verjuice, 24 h	–	–	–
	30% verjuice, 24 h	11.536 ± 0.052^a^	0.948 ± 0.001^a^	9917 ± 2459.317^a^
	70% verjuice, 24 h	11.281 ± 0.458^a^	0.945 ± 0.007^a^	10,100 ± 28.284^a^
Band 13 (12 kDa)	100% verjuice, 24 h	10.386 ± 1.208^a^	0.954 ± 0.013^a^	11,124 ± 2935.907^a^
	No Verjuice, 48 h	–	–	–
	30% verjuice, 48 h	11.849 ± 0.033^a^	0.944 ± 0.001^a^	10,378 ± 1541.493^a^
	70% verjuice, 48 h	10.251 ± 0.847^a^	0.956 ± 0.007^a^	10,886 ± 2225.972^a^
	100% verjuice, 48 h	10.559 ± 0.963^a^	0.951 ± 0.008^a^	11,009 ± 2190.617^a^
	No verjuice, 72 h	–	–	–
	30% verjuice, 72 h	10.719 ± 0.322^a^	0.947 ± 0.016^a^	12,328 ± 2771.859^a^
	70% verjuice, 72 h	10.406 ± 0.198^a^	0.953 ± 0.005^a^	11,499 ± 46.669^a^
	100% verjuice, 72 h	10.310 ± 0.063^a^	0.954 ± 0.008^a^	12,389 ± 1333.603^a^

The increased tenderness with increasing marination time can be also attributed to the desmin proteolysis (MW = 55 kDa) by calpain enzymes (Huff‐lonergan et al., 2010) emerged as a significantly decreased area of desmin band during storage at 4°C. The low activity of μ‐ and m‐calpain in the used muscle and low pHs of marinated samples prohibited desmin from being affected by the verjuice concentration (*p* > .05).

The sensitivity of troponin‐T (MW = 40 kDa) to enzymatic degradation by calpain and cathepsin was revealed by the dependence of band area on both marination time and verjuice concentration. The emergence of a 30‐kDa band in all verjuice‐marinated samples and the increase of band intensity (*p* < .05) with acid concentration confirms troponin‐T degradation into small peptides with the molecular weights of 28 and 30 kDa proved by other researchers (Buts et al., 1986; Taylor et al., 1995) considered as a proper index for meat tenderness (Huff‐lonergan et al., 2010; Kemp et al., 2010). These results are in accordance with those of the shear force. The variations trend of tropomyosin (MW = 35 kDa) was not significant (*p* > .05) confirming that tropomyosin has no impact on meat tenderness (Salm et al., 1983; Yates et al., 1983).

The increased intensity of low molecular weight bands such as myosin light chains (MLC_1_ and MLC_2_ with MW of 24 kDa and 17 kDa, respectively) and troponin I (MW = 21 kDa) increased pronouncedly can be attributed to the breakdown of the high molecular weight protein (Kim et al., 2013). It should be noted that the 21‐kDa band of troponin I appeared but that related to troponin C (19 kDa) was not observed. Moreover, unlike the control, a 12‐kDa band in all marinated samples was observed whose intensity increased significantly (*p* < .05) with increase in both variables resulting from the degradation of high molecular weight proteins.

#### 3.1.9 Sarcomere length

**FIGURE 2:**
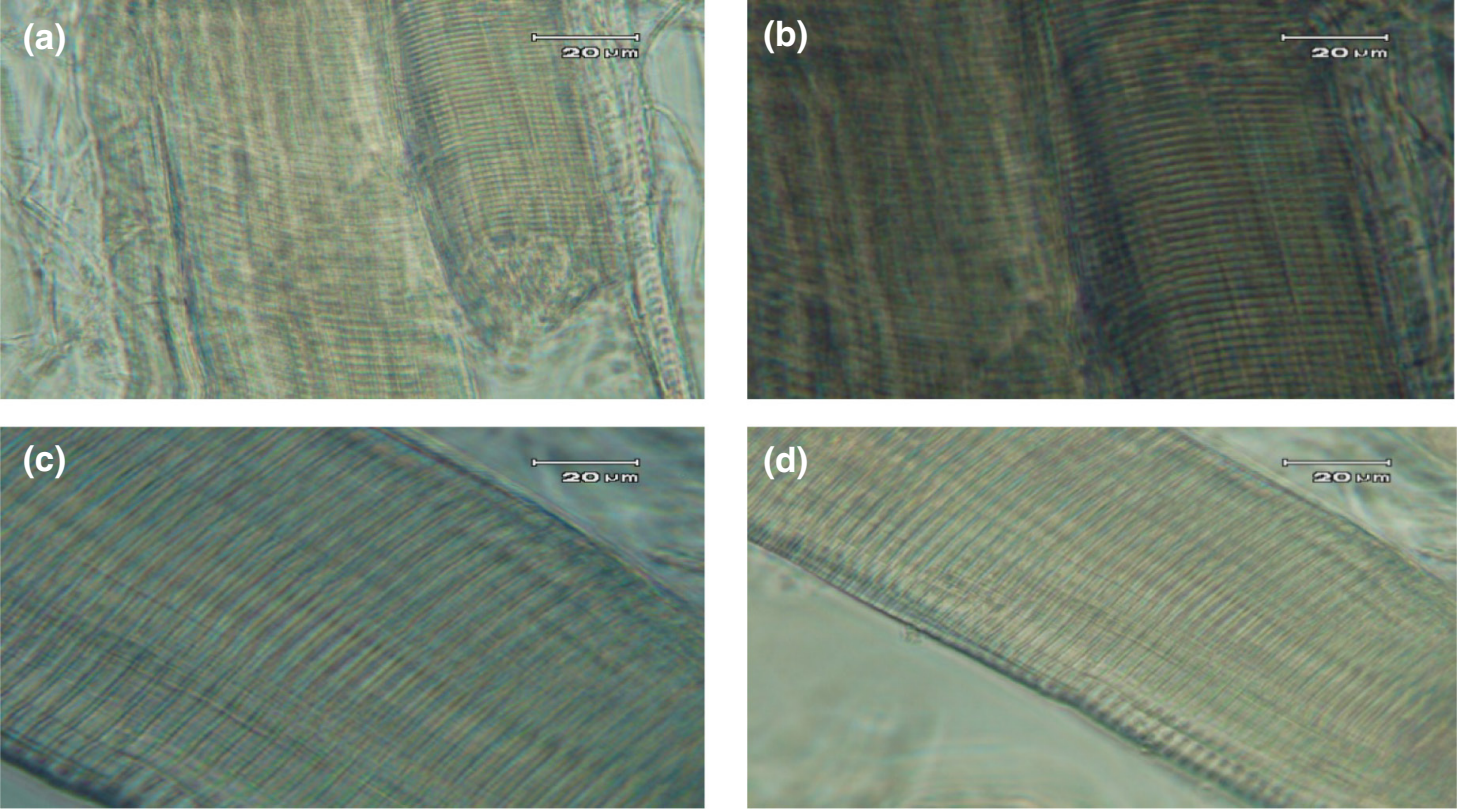
Photographs of myofibrillar proteins from *biceps femoris* beef muscles marinated with %100 verjuice under optical microscopy. (a) untreated control (×40); (b) untreated control (×100); (c) 100% verjuice with 2% w/w sodium chloride for 72 h (×40); (d) 100% verjuice with 2% w/w sodium chloride for 72 h (×100).

The dual effect of acid and salt on myofibrillar proteins, on the one hand, induces the proteolytic weakening of myofibrillar proteins, on the other hand, approaching pH to proteins' IP prevented from observing a regular trend in sarcomere length following increasing acid concentration in the verjuice marinade (Table 2a). As presented in Table 2b, the role of proteolytic enzymes especially cathepsins under acidic conditions was only revealed after 72 h of postmortem storage (*p* < .05). Nevertheless, the increasing trend of sarcomere length with increasing verjuice concentration was only observed for samples marinated until 72 h (Table 3). The pH of these treatments was below proteins' IP at which the development of repulsion forces between protein molecules in myofibrils causes the Z lines to get far from each other and the sarcomere length to increase. Figure 2 compares the light micrographs of unmarinated and marinated muscle fibers of BF for 72 h in 100% verjuice marinade (with magnifications of 40 and 100). There is no evidence of disintegration of Z‐discs by marinating in 100% verjuice solution for 72 h. The constancy and even more intensity of the band corresponding to α‐actinin, which is the major Z‐line protein, on electrophoresis gel is consistent with Z‐line integrity under verjuice marination.

### 3.2 Sensory evaluation of BF marinated with verjuice

**FIGURE 3:**
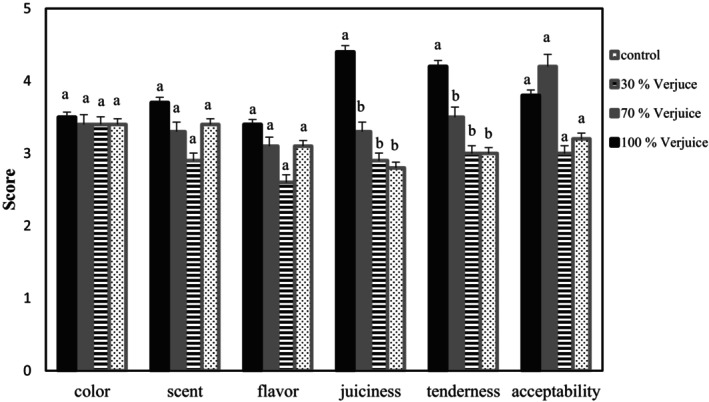
Sensory property evaluation of *biceps femoris* beef muscles marinated with various verjuice levels (0%, 30%, 70%, and 100%) for 48 h.

Sensory evaluation was carried out on beef steaks marinated with all concentrations of verjuice for 48 h. The results revealed that there is no statistically significant difference (*p* > .05) among the scores given to the color, aroma, and flavor of samples marinated with different verjuice concentrations and unmarinated one (Figure 3). The juiciness of samples, known to be of major importance in the overall perception of meats, increased significantly as the verjuice percentage increased. The samples marinated with the highest concentration of acid, despite getting close to IP, tended to have more retention of water during cooking (the lowest cooking loss) and consequently to be juicier.

Meat tenderness was also influenced by acid concentration in the marinade owing to proteolytic weakening of muscle proteins and collagen solubilization so the samples marinated with 100% verjuice were the most tender compared to the other samples (*p* < .05). Other researchers have also reported that meat pieces marinated with acidic solutions (pH < 5) are less tough than control samples (Aktaş & Kaya, 2001; Burke & Monahan, 2003). Although the effect of verjuice solution was not significant on the overall acceptability of samples, the samples marinated with 70% verjuice were most accepted by the panelists and received the highest score.

### 3.3 Organic acid profile of verjuice

#### 3.3.1 Validation of the method applied

The validation of organic acid analysis is shown in Table 6. The linear range of tartaric, malic, oxalic, and lactic acids was found to be 0.1–160 mg/L, 0.01–50 mg/L, 0.01–20 mg/100 g, and 0.05–50 mg/L, respectively. The LOD is used to demonstrate the ability of a method to detect low concentrations of a substance. the LOD of oxalic, tartaric, malic, and lactic acids is 0.01, 0.033, 0.005, and 0.016 mg/L, respectively. The LOD obtained in this study is in agreement with the determination of organic acids by HPLC (Coelho et al., 2018).

**TABLE 6:** Validation parameters for organic acids by RP‐HPLC equipped with an ion exchange column.

Analyzed organic acid	Range of standard (mg/L)	Calibration equation	Correlation coefficient (*R* ^2^)	LOD (mg/L)	Concentration of spiked solution (mg/L)	Found	Recovery (%)
Oxalic acid	0.01–20	*y* = 1E+06*x* + 78,447	*R* ^2^ = .9971	0.01	0.05	0.044 ± 0.00	88
					1	1.05 ± 0.01	105
					10	9.78 ± 1.09	97.8
Tartaric acid	0.1–160	*y* = 22,442*x* + 586,933	*R* ^2^ = .99	0.033	0.05	0.045 ± 0.01	90
					1	1.02 ± 0.03	102
					20	19.48 ± 1.36	97.4
Malic acid	0.01–50	*y* = 539,872*x* + 572,911	*R* ^2^ = .99	0.005	0.01	0.01 ± 0.00	100
					1	0.98 ± 0.00	98
					5	4.5 ± 0.02	90
Lactic acid	0.05–50	*y* = 32,316*x* + 200,486	*R* ^2^ = .9982	0.016	5	4.2 ± 0.03	84
					10	10.11 ± 0.05	101.1
					20	20.37 ± 0.5	101.85

**FIGURE 4:**
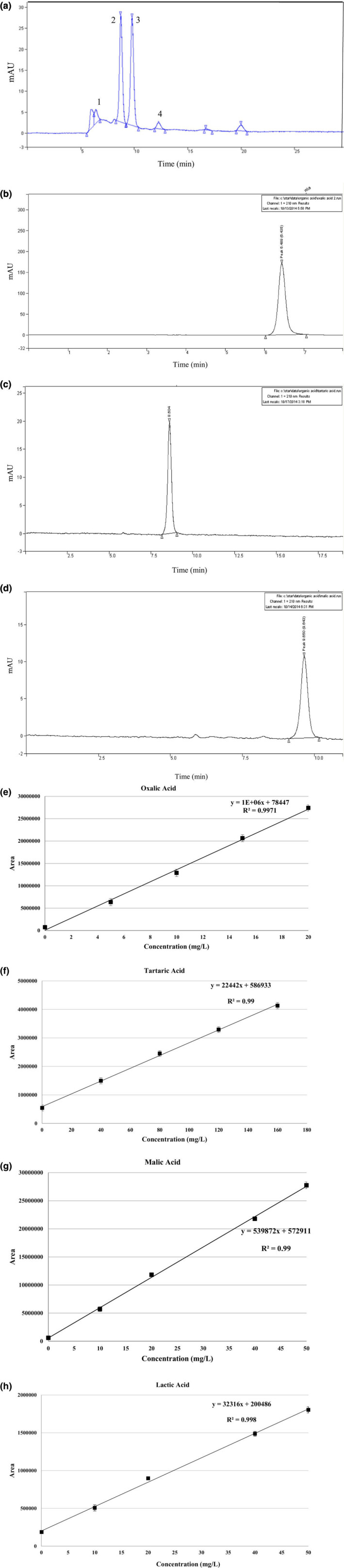
The chromatogram of (a) verjuice organic acids 1 (oxalic acid); 2 (tartaric acid); 3 (malic acid); and 4 (lactic acid), (b) chromatogram of oxalic acid, (c) chromatogram of tartaric acid, (d) chromatogram of malic acid, (e) standard curve of oxalic acid, (f) standard curve of tartaric acid, (g) standard curve of malic acid, (h) standard curve of lactic acid.

HPLC analysis was conducted to elucidate the organic acid profile in the verjuice and also standard curve of organic acids including oxalic, tartaric, malic, and lactic acids used in this study is shown in Figure 4. It can be seen that the dominant acid in the verjuice is tartaric acid 1282.269 mg/100 g (*t*_R_ of 8.64 min) followed by lactic acid 211.974 mg/100 g (*t*_R_ of 12.37 min), malic acid 201.992 mg/100 g (*t*_R_ of 9.68 min), and oxalic acid 28.396 mg/100 g (*t*_R_ of 6.43 min) (Table 7). The presence of phenolic compounds brings about high antioxidant and antimicrobial activity for verjuice in comparison to other acidic marinades which have been used so far. There are various reports on tartaric and malic acid content of different varieties of verjuice. Dupas de Matos et al. (2017) reported the content of tartaric acid and malic (the most dominant organic acids in grapes (90% of the total acids)) in the range of 5.5–14 and 10.9–30.4 g/L, respectively, in the chemical and sensory analysis of six different varieties of unripe grape juice. At the end of the vegetative growth phase, the tartaric acid in unripe grapes can be 15 g/L. Also, the tartaric acid of unripe grape juices ranged from 22.9 to 70.9 g/L (Öncül & Karabiyikli, 2016), 32.7 to 39.8 g/L (Simone et al., 2013), 19.6 to 39.6 g/L (Nikfardjam, 2008), and 24.8 to 30.0 g/L (Hayoglu et al., 2009). Differences in organic acid content are due to genetics, growing region, ecological factors, degree of maturity, and growing year. Also, Shakir and Salih Rashid (2019) in the biochemical and phytochemical profile analysis of unripe black grape juice (verjuice) expressed the content of tartaric acid and malic acid 15.2 and 7.4 mg/mL, respectively. Probably the reason for this slight difference in the content of organic acids was due to the difference in grape variety in the verjuice.

**TABLE 7:** Organic acid profile in the verjuice.

Organic acid	Area	Content (mg/100 g)	Verjuice RT (min)	Standard RT (min)
Oxalic acid	670,910	28.396	6.394	6.428
Tartaric acid	4,828,513	1282.269	8.726	8.638
Malic acid	5,397,121	201.992	9.774	9.801
Lactic acid	382,422	211.974	12.267	12.368

## 4 CONCLUSION

The marination with a combination of 2% NaCl and increasing concentrations of verjuice gave rise to a pH drop in BF muscle. This occurrence was coincident with a pronounced reduction in the shear forces of cooked muscle despite significant decrease in water‐holding capacity. In addition to our prediction on collagen solubilization under acidic conditions, the increased index of myofibrillar fragmentation and the enzymatic proteolysis of myosin and troponin‐T proved through electrophoresis results suggest that tenderization is not caused by swelling of myofibrillar structures stemmed from electrostatic repulsions due to the charge screening effect of salt. Therefore, the abundance, low price, and most importantly the occurrence of polyphenolic compounds in verjuice make it a better and affordable replacer for chemical tenderizers or even herbal proteases. The use of natural meat tenderization methods not only provides a more sustainable and environmentally friendly alternative to chemical tenderizers but also offers health benefits by reducing the intake of artificial additives. Further research could lead to innovative and effective natural meat tenderization solutions that meet consumer demand for high‐quality, tender meat products.

## AUTHOR CONTRIBUTIONS

**Fereshteh Sabzi:** Conceptualization (equal); investigation (equal); methodology (equal); writing – original draft (equal). **Mohammad Javad Varidi:** Conceptualization (equal); funding acquisition (equal). **Mehdi Varidi:** Methodology (equal); supervision (equal); writing – review and editing (equal). **Maryam Asnaashari:** Data curation (equal); formal analysis (equal); software (equal); validation (equal); writing – review and editing (equal).

## CONFLICT OF INTEREST STATEMENT

The authors declare that they have no conflicts of interest.

## Data Availability

The data that support the findings of this study are available on request from the corresponding author. The data are not publicly available due to privacy or ethical restriction.
